# Loneliness in Older Adults with Serious Mental Illness

**DOI:** 10.1093/geroni/igaf122.2784

**Published:** 2025-12-31

**Authors:** Heather Leutwyler, Erin Hubbard

**Affiliations:** University of California, San Francisco, San Francisco, California, United States; University of California, San Francisco, San Francisco, California, United States

## Abstract

**Background:**

Middle-aged and older adults with serious mental illness (SMI) have a lifespan that is about 25 years shorter than persons without a SMI. Tobacco use and limited social networks likely contribute to this premature mortality. Between 70-85% of people living with SMI currently smoke cigarettes. Social disconnection and limited social networks are common in people with SMI. To understand how loneliness is associated with tobacco use and symptoms among middle-aged and older adults with SMI, we report a cross-sectional analysis from the baseline assessment of a sample of middle-aged and older adults with SMI enrolled in a smoking cessation study.

**Methods:**

We analyzed baseline data from 64 participants with SMI > 40 (mean age 53.7, age range 40-73) enrolled in a smoking cessation program. We assessed loneliness with the 3-item UCLA loneliness scale, cigarette use with participants’ self-report of the number of cigarettes smoked per week, and psychiatric symptoms with the Brief Psychiatric Rating Scale.

**Results:**

We identified significant positive associations between the higher ratings on the item “feeling left out” on the loneliness scale and a higher number of cigarettes smoked in the past week (p=.03). We identified a significant positive association between total psychiatric symptom score and higher ratings on the item “feeling left out” (p=.002).

**Conclusion:**

Results suggest daily cigarette use is associated with higher self-ratings of loneliness, and higher symptom burden is associated with higher self-ratings of loneliness. Future work could test integrating social connections to better support middle aged and older adults with SMI.

